# Supplementary material for: Learning curve and surgical outcome of robotic assisted colorectal surgery with ERAS program
Source: Sci Rep. 2022 Nov 29;12:20566. doi: 10.1038/s41598-022-24665-w (PMC9709162; doi:10.1038/s41598-022-24665-w)
Supplement: Supplementary file 2 — Supplementary Information 2. [file 41598_2022_24665_MOESM2_ESM.zip › RAL Raw data-Ñ[▒K/141/HS_ñw╝╨░O▒Kñσ.pdf]

正式摘要(電子簽章：000002343110048)
W52 / W52 英文標題(注意:無英文姓名-請補病人基本檔!) 【讀取】憑證

臺中榮民總醫院

出院病歷摘要

轉入號： 轉出號：

[redacted]

(9)入院日期	111/01/06 (2022) 直腸外科 W52 - 072
(10)轉科日期	
(11)出院日期	111/01/11 (2022) 住院天數計 5 日

診斷

- 入院 :1. Rectum cancer
  2. HTN
- 出院 :1. Rectum cancer, s/p Robotic assist LAR + loop ileostomy on 2022/01/07
  2. HTN

主訴

- Bloody stool in 2021/11

病史

- This is a 76 years old male patient with history of

  1. HTN

  According to the patient, he suffered form bloody stool in 2021/11. He visited FongYuan H. and colonscopy with biopsy was done there, showed adenocarcinoma of colon, 10cm FAV. Abdominal CT was done there, showed no obvious distant metastasis. For personal reason, he visited our CRS OPD for second opinion. After discussion, this time he was admitted to our ward for scheduled operation.

體檢發現

- Anal inspection:
  Recto digital examination:
  Sigmoidoscopy:
  Anoscopy:
  Abdomen:
  - General appearance :
    - easy looking , conscious clear , without cardiopulmonary distress
    - Vital signs自動帶入date 體溫(BT) 脈搏(P) 呼吸(R) 血壓(BP) 身高(BH) 體重(BW) BMI
      20220106 36C 81/min 18/min 137/88mmHg 158.5cm 67.6Kg 26.9
    - Vital signs: B.P. 137/88 mmHg , P.R. 81 /min , R.R. 18 /min , B.T. 36 ℃
  - HEENT :
    - Eyes: no anemic conjunctiva, no icteric sclera, normal EOM
    - ENT: no otorrhea, no rhinorrhea, no erythematous mucosa, no injected tonsil, no deformity
  - Neck: supple, freely movable, trachea midline,no deformity, no engorged jugular vein , no palpable thyroid or lymph node enlargement
  - Chest:
    - Inspection: Grossly normal, symmetric and fully expansion, no accessory muscle used, no operation scar, no spider angiomata, no gynecomastia
    - Palpation: no palpable mass or nodule, no subcutaneous emphysema, no tenderness
    - Ausculation: clear breathing sound
  - Heart:
    - Palpation: no thrill, no heave
    - Percussion: normal heart size
    - Ausculation: regular heart beat, normal S1, S2, no gallop, no murmur
  - Extremities: No deformity, full ranged of motion without limitation, no clubbing finger or nail cyanosis, no pitting edema, no ulcer or pigmentation, no obvious skin lesion, no varicose vein, no bone, joint or muscle tenderness

  CRS CONDITION
  - Karnofsky performance status: (100) Normal, no complaints, no evidence of disease
  - PE of Abdomen:
  - CEA: 2.46 ng/ml
  - Imaging Studies:
  - Colonoscope report: .
  - Figures:
    1. colon & rectum

手術日期及方法

- 手術日期及方法20220107 Robot Assisted Low Anterior Resection + loop ileostomy
- 手術結果及發現 :
  - 手術日期: 1110107 麻醉方式:
    手術名稱:Robot Assisted Low Anterior Resection + loop ileostomy
    術前診斷:Malignant tumor of rectum 8CM
    術後診斷: Rectal tumor at middle rectum

住院治療經過

- After admission, he received pre-OP scopy with ink localization and Robotic assist LAR + loop ileostomy on 2022/01/07. He start try bland II diet since 01/09.Foley was removed once but re-on due to AUR. Under relative stable condition, we remove Foley again on 1/11, and self voiding noticed. Then, he discahrged on 1/11, arrange OPD follow up.

合併症

一般檢查

- 累積報告 :

DATE	TIME	ALB	TP	BIL.T	BIL.D	ALKP	AST	ALT	LDH	AMY	LIPASE	NH3	Ferritin	UA
1110107	0845	4.4	6.8	1.05	0.23	97	33	30						4.7

1110107	2139	3.6		0.70	0.18	74	27	25						

DATE	TIME	WBC	RBC	HGB	HCT	MCV	PLT	BLST	NEUT	BAND	SEG	LYM	MON	EOS	BAS
1110107	0907	4880	4.14	13.7	40.9	98.8	223		54.3			31.6	9.6	3.5	1.0
1110107	2139	10320	3.34	11.0	33.1	99.1	185		90.1			6.6	3.2	0.0	0.1

DATE	TIME	NA	K	CL	BUN	CREAT	eGFR	CA	Mg	P	CRP	ESR	PCT	D-Dimer	LACTATE
1110107	0845	138	3.8		16	1.15	65.71				0.155				
1110107	2139	140	3.8	107	18	1.25	59.69	8.3							

DATE	TIME	GLUCOSE	HbA1C	CHOL	TG	HDL	LDL	KETONE	CK	CKMB	TRO-T	PT-P	APTT-P	NT-pro BNP
1110107	0845	90		185	104	41	124							
1110107	2139	127												

特殊檢查

病理報告

其它

- **檢驗項目 :Sigm 報告日期 :1110107**

Result:

年齡：

內視鏡型號： olympus290-20 檢查開始時間： 1200 檢查至盲腸時間： 檢查結束時間： 1220

適應症(Indication)： 其他臨床目的施行之大腸鏡 CRC

大腸鏡伸入位置(Insertion level)最深位置： Sigmoid colon (距離肛門口 公分)

未達盲腸原因：(複選) N N

Y N N

檢查前用藥(Premedication)：

止痙攣藥物：

無

止痛鎮靜藥物：

N N N

N N

清腸用藥(Colon cleansing agent)：(複選)

N N N N N N

N

清腸給藥時間(Preparation time)： Split dose

清腸程度(Colon cleansing level)： 良好(Excellent)

抗凝血藥物(Anti platelet/Coagulant)： 無

Y N N

大腸鏡檢後併發症(Complication)：(複選)

N N

檢體總數： 0 顆(含增生性瘜肉) 臨床診斷為C2(腺瘤)或C3(其他瘜肉)或D(腫瘤)應逐顆呈現

若有2個以上診斷結果，填寫順序依序為4大腸癌＞3瘜肉＞2潰瘍性大腸炎＞1痔瘡＞5其他>0正常

檢查結果(Findings)：

大腸癌

Colonscopy was up to S-colon. Tumor was noted at 15cm FAV, s/p tatto.

Colon tumor

診斷：

其他：

植入物國際條碼：

病灶處未處置原因：(複選)

N

N

N

N

N N

N

N

病灶未送病理原因：

註1:(若病灶不止一個，請再自行增加欄位)。

註2:若臨床診斷(內視鏡診斷)診斷為「增生性瘜肉」，得無須送驗檢體；惟，需算在總顆數內。

註3:無病灶：如完全正常，至少應附8張照片(盲腸、升結腸、肝彎曲、橫結腸、脾彎曲、降結腸、乙狀結腸、直腸)；如有病灶應附病灶照片。(照片無須上傳，留於醫院存查)

註4:清腸程度

良好(Excellent)：僅有少量的清澈糞水，且95%以上的腸道黏膜可被清楚觀察

適當(Good)：較多量的清澈糞水佔據小於25%的黏膜，且90%以上的腸道黏膜可被清楚觀察

尚可(Fair)：半固體的糞便可被清除，清洗後90%以上的腸道黏膜可被清楚觀察

不良(Poor)：半固體的糞便無法被清除，且不到90%的腸道黏膜可被清楚觀察

註5:併發症Significant bleeding 瘜肉切除後發生出血情形：

(1) 中度(Moderate):達輸血治療者。

(2) 重度(Severe): 接受介入性治療者，例如：經血管攝影栓塞、外科手術等。

第1個病灶

臨床診斷(內視鏡診斷)：

病灶處位置： 臨床診斷為C2(腺瘤)或C3(其他瘜肉)或D(腫瘤)，病灶處位置應為單選；其餘(非C2,C3,D)得複選

N N N N N

N N N N

N N N N N (距離肛門口 公分)

病灶處處置：(複選)

N N N N

N

N

N N N

N N N N

N N

N N

病灶處有無檢體： (無檢體以下無需填寫)

檢體大小(內視鏡下大小)： 公分(小數點1位) 檢體編號： (以A到Z呈現，區分每一檢體)

第2個病灶

- ---
臨床診斷(內視鏡診斷)：
病灶處位置： 臨床診斷為C2(腺瘤)或C3(其他瘜肉)或D(腫瘤)，病灶處位置應為單選；其餘(非C2,C3,D)得複選
N N N N N
N N N N
N N N N N (距離肛門口 公分)
病灶處處置：(複選)
N N N N
N N
N
N N
N N N
N
N
N N
病灶處有無檢體：
(無檢體以下無需填寫)
檢體大小(內視鏡下大小)：
公分(小數點1位) 檢體編號： (以A到Z呈現，區分每一檢體)

第3個病灶

臨床診斷(內視鏡診斷)：
病灶處位置： 臨床診斷為C2(腺瘤)或C3(其他瘜肉)或D(腫瘤)，病灶處位置應為單選；其餘(非C2,C3,D)得複選
N N N N
N
N N N N
N N N N N (距離肛門口 公分)
病灶處處置：(複選)
N N N N
N N
N N
N
N N
N N
N
N
N N
病灶處有無檢體：
(無檢體以下無需填寫)
檢體大小(內視鏡下大小)：
公分(小數點1位) 檢體編號： (以A到Z呈現，區分每一檢體)

第4個病灶

臨床診斷(內視鏡診斷)：
病灶處位置： 臨床診斷為C2(腺瘤)或C3(其他瘜肉)或D(腫瘤)，病灶處位置應為單選；其餘(非C2,C3,D)得複選
N N N N N
N N N N
N N N N N (距離肛門口 公分)
病灶處處置：(複選)
N N N N
N N
N N
N
N
N N
N
N
N
N N
病灶處有無檢體： (無檢體以下無需填寫)
檢體大小(內視鏡下大小)： 公分(小數點1位) 檢體編號：
(以A到Z呈現，區分每一檢體)

第5個病灶

臨床診斷(內視鏡診斷)：
病灶處位置： 臨床診斷為C2(腺瘤)或C3(其他瘜肉)或D(腫瘤)，病灶處位置應為單選；其餘(非C2,C3,D)得複選
N N N N N
N N N N
N N N N N (距離肛門口 公分)
病灶處處置：(複選)
N N N N
N N
N N
N
N
N N
N
N
N
N N
病灶處有無檢體： (無檢體以下無需填寫)
檢體大小(內視鏡下大小)： 公分(小數點1位) 檢體編號： (以A到Z呈現，區分每一檢體)

其他病灶

出院時情況

- 3.改門診治療

出院指示

藥名	劑量	劑量單位	用法	頻次	數量	數量單位	現況	天數	單位
Estazolam tab 2mg	1	TAB	PO	HSPRN	7	TAB	TH	7	大
Ultracet tab	1	TAB	PO	BIDPRN	14	TAB	TH	7	天
OCAS Harnalidge 0.4 mg	1	TAB	PO	QDAC	7	TAB	TH	7	天
(口)Takepron OD tab 30mg	1	TAB	PO	QDAC	7	TAB	TH	7	天

出院計劃

- 門診日期
  - date on 2022/01/18

主治醫師 2062F 蔣鋒帆 / 住院醫師 2339C 蔡濬愇

正式摘要(電子簽章：000000234311048)

W52 / W52

doctorRank:B
